# Quantifying variation in *δ*^13^C and *δ*^15^N isotopes within and between feathers and individuals: Is one sample enough?

**DOI:** 10.1007/s00227-015-2618-8

**Published:** 2015-02-15

**Authors:** W. James Grecian, Rona A. R. McGill, Richard A. Phillips, Peter G. Ryan, Robert W. Furness

**Affiliations:** 1 Institute of Biodiversity, Animal Health and Comparative Medicine, College of Medical, Veterinary and Life Sciences, Graham Kerr Building, University of Glasgow, Glasgow, G12 8QQ Scotland, UK; 2 NERC Life Sciences Mass Spectrometry Facility, Scottish Universities Environmental Research Centre, Rankine Avenue, East Kilbride, G75 0QF Scotland, UK; 3 British Antarctic Survey, Natural Environment Research Council, High Cross, Madingley Road, Cambridge, CB3 0ET UK; 4 DST/NRF Centre of Excellence, Percy FitzPatrick Institute, University of Cape Town, Rondebosch, 7701 South Africa

## Abstract

Studies of avian migration increasingly use stable isotope analysis to provide vital trophic and spatial markers. However, when interpreting differences in stable isotope values of feathers, many studies are forced to make assumptions about the timing of moult. A fundamental question remains about the consistency of these values within and between feathers from the same individual. In this study, we examine variation in carbon and nitrogen isotopes by sub-sampling feathers collected from the wings of adults of two small congeneric petrel species, the broad-billed *Pachyptila vittata* and Antarctic prion *P. desolata*. Broad-billed prion feather vane material was enriched in ^15^N compared to feather rachis material, but there was no detectable difference in *δ* ^13^C. Comparison of multiple samples taken from Antarctic prion feathers indicated subtle difference in isotopes; rachis material was enriched in ^13^C compared to vane material, and there were differences along the length of the feather, with samples from the middle and tip of the feather depleted in ^15^N compared to those from the base. While the greatest proportion of model variance was explained by differences between feathers and individuals, the magnitude of these within-feather differences was up to 0.5 ‰ in *δ* ^15^N and 0.8 ‰ in *δ* ^13^C. We discuss the potential drivers of these differences, linking isotopic variation to individual-level dietary differences, movement patterns and temporal dietary shifts. A novel result is that within-feather differences in *δ* ^13^C may be attributed to differences in keratin structure within feathers, suggesting further work is required to understand the role of different amino acids. Our results highlight the importance of multiple sampling regimes that consider both within- and between-feather variation in studies using stable isotopes.

**Electronic supplementary material:**

The online version of this article (doi:10.1007/s00227-015-2618-8) contains supplementary material, which is available to authorized users.

## Introduction

Stable isotope analysis has provided the study of avian migration with vital trophic and spatial markers (Hobson 1999; Newsome et al. 2007; Inger and Bearhop 2008). The isotopic composition of animal tissues reflects the diet of the individual during tissue formation, and so, as feather keratin is metabolically inert after synthesis, the isotopic values of feathers provide information on the diet and location of an individual during the moulting period (Hobson and Clark 1992a; Bearhop et al. 2002). Knowledge of the sequence and timing of moult in adult birds is, therefore, essential for robust interpretation of stable isotope data from feathers (Bridge 2006; Catry et al. 2013; Carravieri et al. 2014). Although moult occurs primarily during the nonbreeding period, some species and particularly birds that have failed breeding may replace part of the plumage at the breeding grounds and then may suspend moult until birds reach the nonbreeding region (Bridge 2006; Ramos et al. 2009a; Catry et al. 2013; Crossin et al. 2013).

The isotopic signatures of animal tissues have been used to identify foraging locations (Jaeger et al. 2010) and to infer the migratory patterns of seabirds and marine mammals in the Southern Ocean (Best and Schell 1996; Cherel et al. 2009; Quillfeldt et al. 2010). Combining isotopic signatures of feathers with knowledge of moult patterns has allowed the reconstruction of migration routes (Ramos et al. 2009a), provided information on the possible transmission of the H5N1 strain of avian influenza (Chang et al. 2008; Horacek 2011), and revealed variation in habitat preferences both within and between species during the nonbreeding period (Cherel et al. 2006; Phillips et al. 2009). This has enabled the migratory patterns and habitat selection of individuals during the winter to be linked to subsequent condition and breeding performance (Marra et al. 1998; Furness et al. 2006; Inger et al. 2008) and identifies the geographical origins of pollutant burdens carried by seabirds (Leat et al. 2013).

Many studies make assumptions about the timing of moult when interpreting differences in stable isotope values of feathers, yet fundamental questions remain about the consistency of these values within and between feathers from the same individual, and how to disentangle the internal and external sources of isotopic variation. This is a particular issue for species in which moult may be prolonged or the timing poorly defined, including those that move between several nonbreeding areas. Temporal shifts in space use or diet are likely to result in isotopic shifts both within-individual feathers, and between feathers along the feather tract (Inger and Bearhop 2008). Furthermore, isotopic signatures provide only indirect evidence of habitat use or foraging preferences (Authier et al. 2012), and isotopic variation among prey species sampled from similar locations makes linking individuals to specific areas difficult (Schmidt et al. 2003). Individual-level differences in *δ* ^15^N may be the result of dietary differences, reflecting either the availability of prey encountered by an individual, or individual dietary specialisation (Bolnick et al. 2003; Ceia et al. 2012; Patrick et al. 2014), and would lead to highly consistent isotope values within-individual tissues. Nevertheless, variation in *δ* ^15^N associated with concurrent variation in *δ* ^13^C is more likely to be associated with changes in both prey and baseline levels due to habitat differences. Differences may also arise due to differences in structure or protein composition within feathers or the seasonal storage of nutrients for feather synthesis (Fox et al. 2009). It is important, therefore, to assess the degree of intra- and inter-feather variation in isotope values to aid in their interpretation (Wassenaar and Hobson 2006; Jaeger et al. 2009; Cherel et al. 2009).

In this study, our aim was to explore variation in carbon and nitrogen isotope values within and between feathers of individual birds, and the factors that contribute to such variation. We examined isotopic variation in feathers collected from adults of two small congeneric petrel species, the broad-billed prion *Pachyptila vittata* and Antarctic prion *P. desolata*. Broad-billed prions are dietary specialists that feed predominantly on large copepods, and Antarctic prions feed on a wider range of small zooplankton taxa (Brooke 2004). Both species are vulnerable to predation by skuas *Stercorarius* spp. that breed near to prion colonies (Furness 1987), providing the remains of recently killed birds for sampling. These two species were considered to be ideal for our study, as narrow diet breadth should limit the degree of variation in feather isotope values related to individual diet specialisation. In addition, prions undergo a complete moult of primary feathers during the nonbreeding period (Bridge 2006), allowing us to examine changes in isotope values within and between feathers of individuals.

## Materials and methods

### Sampling protocol

**Fig. 1:**
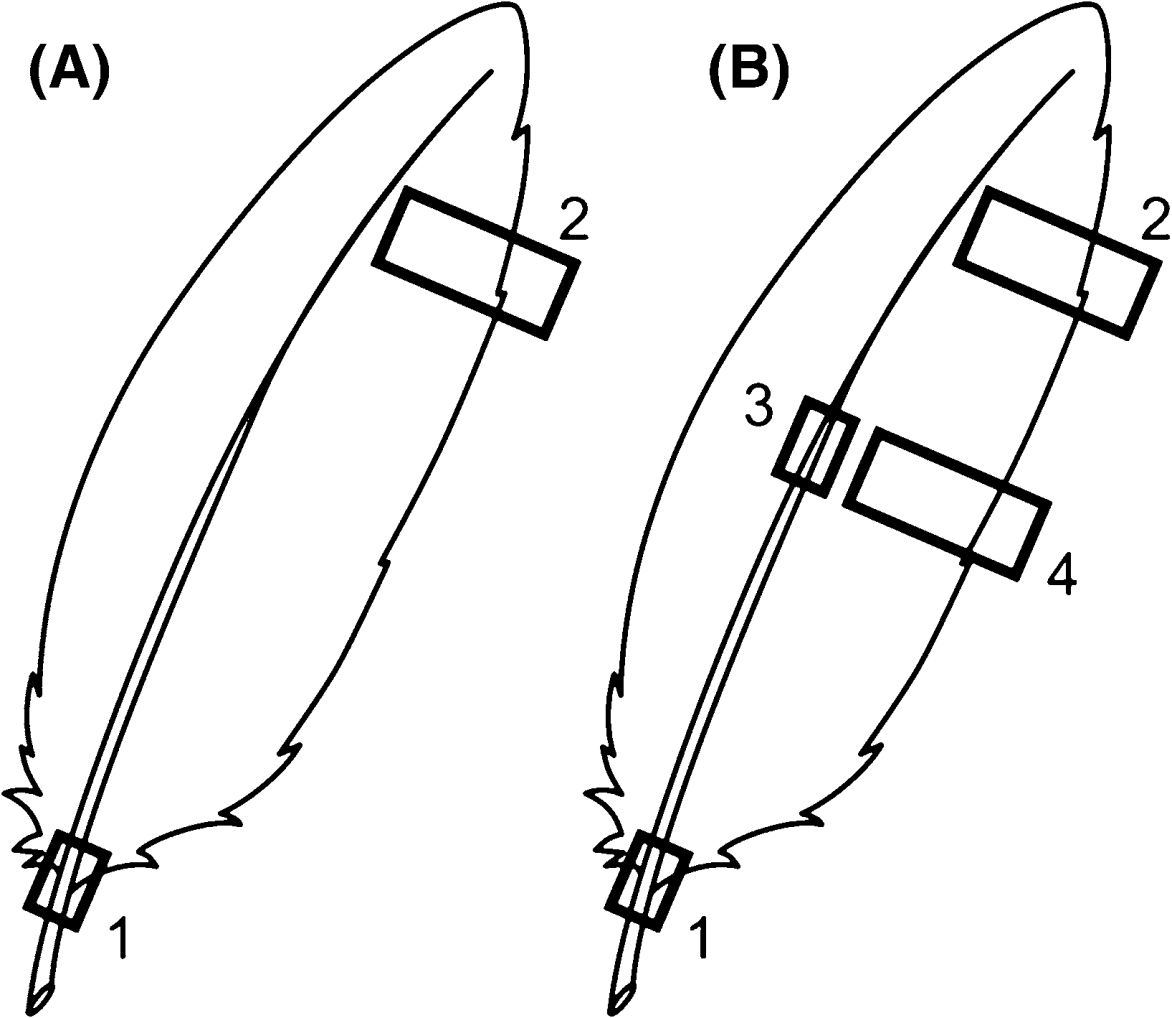
Feather sampling protocols for **a** broad-billed prion and **b** Antarctic prion, illustrating the sampling location of (*1*) rachis, (*2*) vane, (*3*) mid-rachis and (*4*) mid-vane

In the austral summer of 2010/2011, the remains of 15 adult broad-billed prions were collected from skua territories on Gough Island (40°19′S, 9°56′W). To examine variation within the wing, three primary feathers (P2, P5 and P9) and one primary covert feather were sampled from the wings of all 15 birds. To examine differences within feathers, we took one small sample from the inner vane at the tip of the feather, and one from the intersection between rachis and calamus at the feather base (Fig. 1a). In the austral summer of 2011/2012, one primary feather (P5) was collected from the wings of 11 adult Antarctic prions collected in skua territories on Bird Island, South Georgia (54°00′S, 38°03′W). To examine potential structural differences within feathers, we took four samples from each feather: parts of the rachis from the base and middle of the feather, and inner vane from the middle and tip of the feather (Fig. 1b). All vane material was taken from paler sections of the feather to limit the potential effect of melanin on the bulk isotopic signature (Michalik et al. 2010), and base rachis material was sampled at the intersection of the rachis and calamus at the start of the vane to prevent contamination by blood. For analysis, feather material was cut into small fragments using stainless steel scissors and ~0.7 mg aliquots weighed into a tin cup.

### Isotope analysis

Analysis of feather stable carbon and nitrogen isotopes was conducted at the East Kilbride Node of the Natural Environment Research Council Life Sciences Mass Spectrometry Facility via continuous flow isotope ratio mass spectrometry, using a Costech (Milan, Italy) ECS 4010 elemental analyser interfaced with a Thermo Scientific (Bremen, Germany) Delta V plus mass spectrometer. Stable isotope ratios are reported in *δ* notation, expressed as parts per thousand (‰) deviation according to the equation *δX* = [(R_sample_/R_standard_) − 1], where *X* is ^13^C or ^15^N, R is the corresponding ratio ^13^C/^12^C or ^15^N/^14^N, and R_standard_ is the ratio of the international references VPDB for carbon and AIR for nitrogen. The measurement precision, calculated as the standard deviation associated with repeated analyses of internal standards (tryptophan), was ± 0.123 ‰ for *δ* ^13^C and ± 0.295 ‰ for *δ* ^15^N.

### Statistical analysis

Differences in feather isotope values between samples were examined by fitting isotope value as the response variable in a linear mixed-effects model with sample type included as a two-level (rachis/vane) or four-level (rachis/mid-rachis/mid-vane/vane) fixed effect. We fitted separate models for carbon and nitrogen isotopes, and for each species. As models for broad-billed prions included two samples per feather, and four feathers per bird, we fitted feather type and bird identity as nested random intercepts. Models for Antarctic prions included data from four samples per feather, but only one feather per bird. In this case, we fitted bird identity as a random intercept.

Models were fitted using the package lme4 version 1.0 (Bates et al. 2014) in R version 3.0.2 (R Core Team 2014). To test the effect of sample type on feather isotopes, models were first fitted with maximum likelihood (ML) and the full model compared against an intercept-only model using likelihood ratio tests (LRTs). Models were then refitted using restricted maximum likelihood (REML) to estimate effect sizes. We tested the significance of bird identity (Bird ID) and feather type (Feather ID) as random effects by comparing models with and without each term against a model with both terms using restricted likelihood ratio tests (RLRT) fitted with the R package RLRsim (Scheipl et al. 2008). For Antarctic prions, where sample was a four-level factor, we conducted post hoc comparisons by calculating differences between least-squares means with Satterthwaite’s approximation for degrees of freedom, using the R package lmerTest (Kuznetsova et al. 2014). To provide an estimate of model fit, we extracted final model variance components for all models. Following Nakagawa and Schielzeth (2013), we present the proportion of variance explained by the fixed effects as the marginal *R* ^2^ (*R*_GLMM(*m*)_^2^) and the proportion of variance explained by the whole model (fixed + random) as the conditional *R* ^2^ (*R*_GLMM(*c*)_^2^). Unless indicated otherwise, means are provided ±SD.

## Results

### Broad-billed prion

**Fig. 2:**
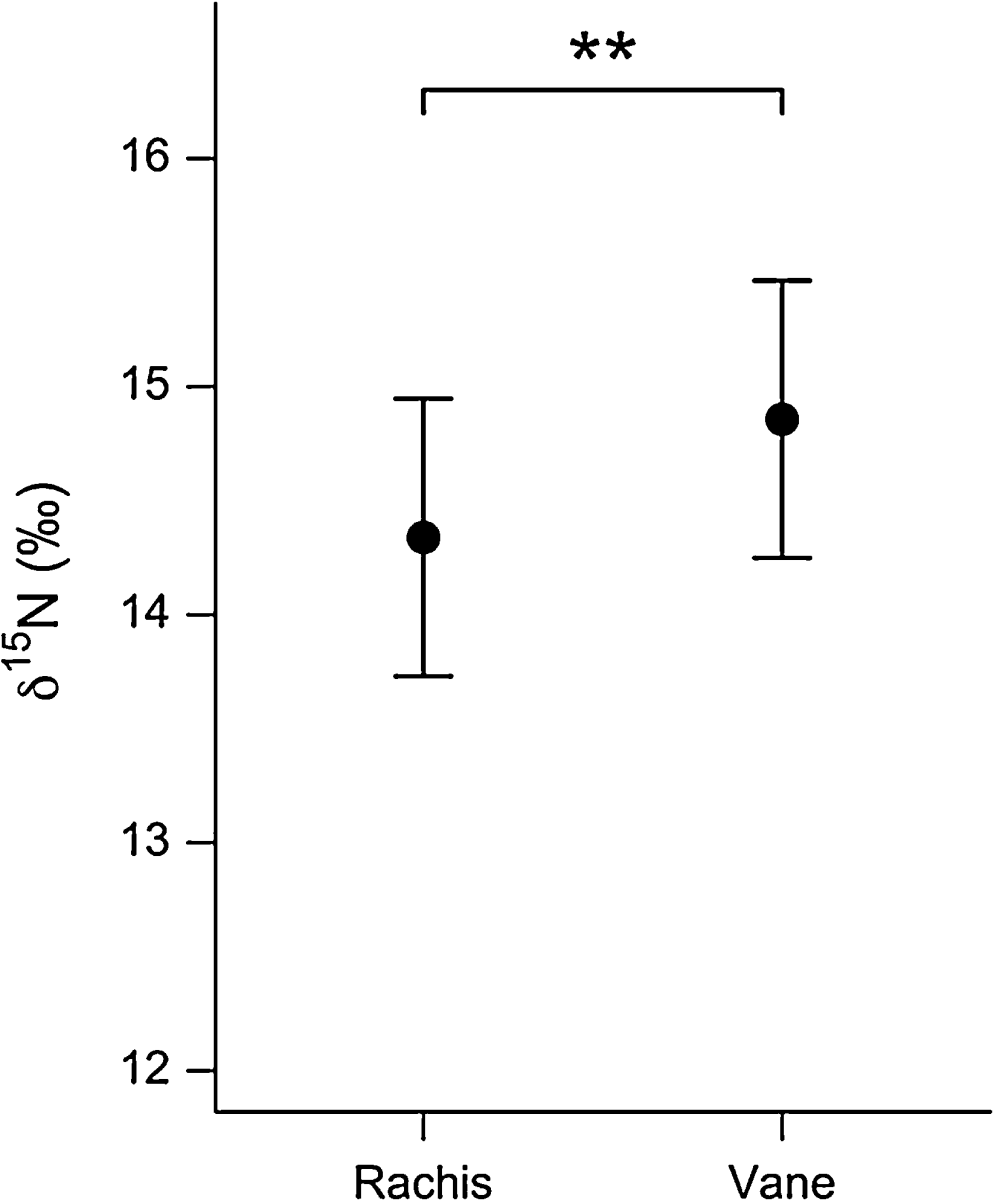
Difference in *δ* ^15^N between broad-billed prion feather vane and rachis material (*χ* ^2^ = 7.209, *P* = 0.007). Values are model predictions (mean ± SE) from a linear mixed-effects model with bird identity and feather type as random intercept terms (** *P* < 0.01)

Vane and rachis samples differed in *δ* ^15^N (*χ* ^2^ = 7.38, *P* = 0.007), with values in the vane an estimated 0.52 ‰ higher than those in the rachis (14.34 ‰, Fig. 2), and the difference in *δ* ^13^C of vane and rachis samples taken from broad-billed prions bordered on significant (*χ* ^2^ = 3.84, *P* = 0.050). Summary data can be found in Table 1. Differences between samples explained a very small amount of variation in the *δ* ^15^N model (*R*_GLMM(*m*)_^2^ = 0.8 %), and variation in *δ* ^15^N was instead explained by the combination of Bird ID (RLRT = 23.66, *P* < 0.001) and Feather ID (RLRT = 40.73, *P* < 0.001) in the random-effects structure of the model (*R*_GLMM(*c*)_^2^ = 87.6 %, Table 2). Variation in *δ* ^13^C among broad-billed prion samples was better explained with the inclusion of Bird ID (RLRT = 5.24, *P* = 0.008) and Feather ID (RLRT = 7.01, *P* = 0.003) in the random-effects structure, and the final model explained approximately half the observed variation in *δ* ^13^C (*R*_GLMM(*c*)_^2^ = 48.4 %, Table 2).

**Table 1:** Summary of feather *δ* ^13^C and *δ* ^15^N values for broad-billed and Antarctic prions included in the study

	*δ* ^13^C (‰)			*δ* ^15^N (‰)		
	Min	Max	Mean ± SD	Min	Max	Mean ± SD
Broad-billed prion (*n* = 15)						
P2						
Rachis	−18.2	−16.6	−17.1 ± 0.5	8.1	18.2	15.0 ± 3.5
Vane	−19.4	−16.3	−17.3 ± 1.0	7.9	18.5	14.8 ± 3.5
P5						
Rachis	−18.9	−15.7	−17.2 ± 0.8	8.0	17.8	14.7 ± 2.7
Vane	−18.2	−16.4	−17.2 ± 0.5	8.5	19.1	15.2 ± 3.2
P9						
Rachis	−18.8	−17.0	−17.9 ± 0.4	11.5	15.2	12.9 ± 1.1
Vane	−18.1	−16.1	−17.3 ± 0.5	12.1	16.4	14.1 ± 1.0
Covert						
Rachis	−18.4	−16.1	−17.2 ± 0.6	7.7	18.8	14.7 ± 2.7
Vane	−17.9	−16.2	−16.9 ± 0.6	8.1	18.7	15.3 ± 3.6
Antarctic prion (*n* = 11)						
P5						
Rachis	−22.7	−19.0	−21.2 ± 1.1	10.1	11.4	10.6 ± 0.4
Mid-rachis	−23.0	−19.2	−21.2 ± 1.0	9.5	11.1	10.2 ± 0.4
Mid-vane	−24.9	−19.2	−21.7 ± 1.5	9.7	11.7	10.3 ± 0.5
Vane	−24.5	−19.5	−21.9 ± 1.3	9.9	11.2	10.3 ± 0.4

**Table 2:** Variance components from linear mixed-effects models testing the effect of sample location on the isotopic ratios of broad-billed (BBP) and Antarctic prion (AP) feathers

	*R*_GLMM(*m*)_^2^	Feather ID	Bird ID	*R*_GLMM(*c*)_^2^	Residual
Broad-billed prion (*n* = 15)					
*δ* ^15^N	0.008	0.324	0.545	0.876	0.124
*δ* ^13^C	–	0.280	0.204	0.484	0.516
Antarctic prion (*n* = 11)					
*δ* ^15^N	0.119	–	0.511	0.630	0.370
*δ* ^13^C	0.075	–	0.749	0.824	0.176

Marginal *R* ^2^ (*R*_GLMM(*m*)_^2^) represents the proportion of variance explained by the fixed effects, and conditional *R* ^2^ (*R*_GLMM(*c*)_^2^) the proportion of variance explained by the whole model (fixed + random effects). BBP models included both feather type (Feather ID) and bird identity (Bird ID) as random intercept terms, AP models only included samples from one feather and so Bird ID was included as a random intercept

### Antarctic prion

We detected significant differences between the *δ* ^15^N values of the four samples taken from the P5 feathers of Antarctic prions (*χ* ^2^ = 11.45, *P* = 0.010, *R*_GLMM(*m*)_^2^ = 11.9 %). The *δ* ^13^C values of the four samples taken from P5 also differed (*χ* ^2^ = 14.36, *P* = 0.002, *R*_GLMM(*m*)_^2^ = 7.5 %). Nevertheless, including Bird ID in the random-effects structure explained a greater proportion of variation in both *δ* ^15^N (RLRT = 14.37, *P* < 0.001, *R*_GLMM(*c*)_^2^ = 63.0 %, Table 2) and *δ* ^13^C (RLRT = 33.69, *P* < 0.001, *R*_GLMM(*c*)_^2^ = 82.4 %, Table 2).

**Fig. 3:**
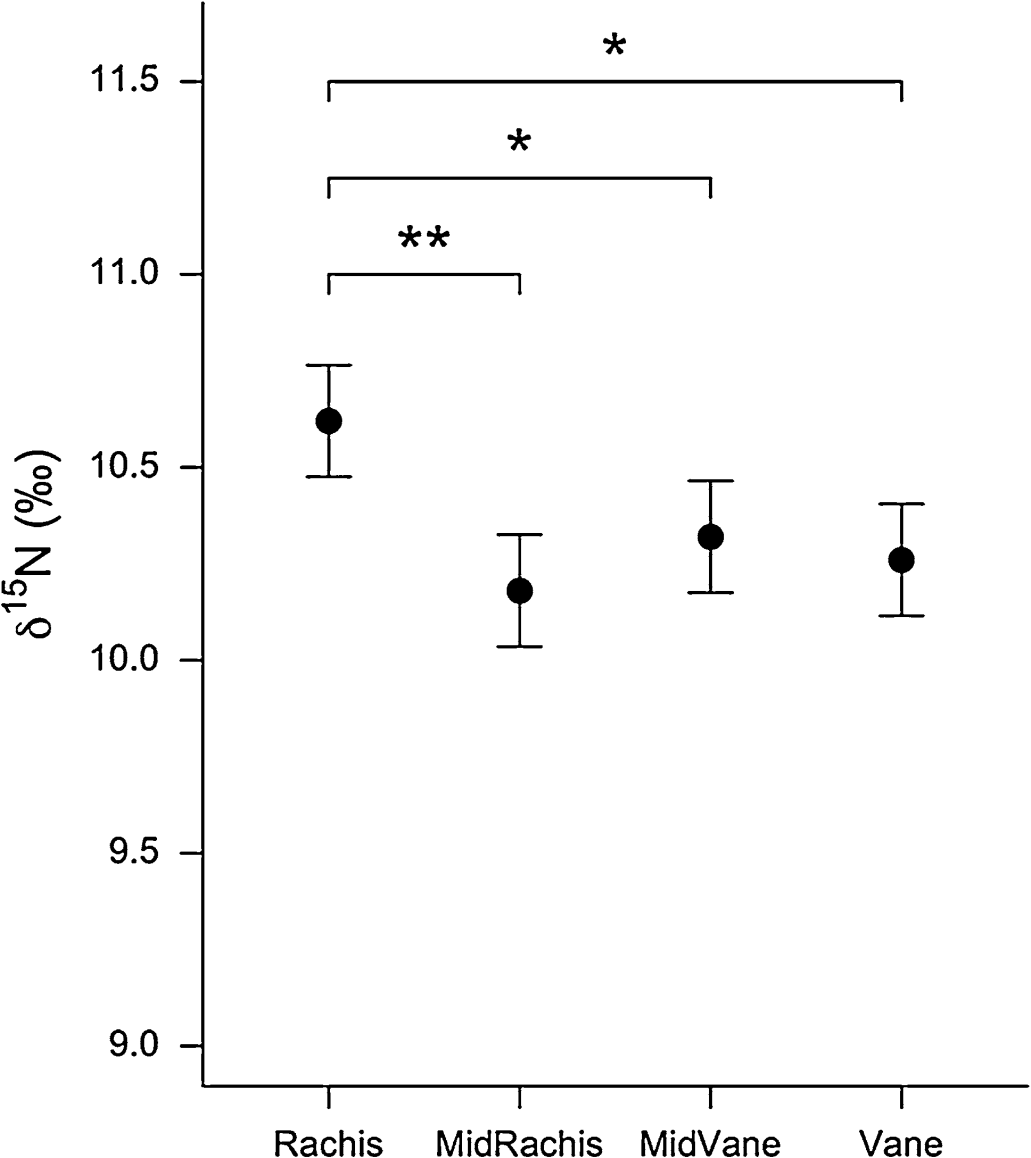
Post hoc comparison of the differences in Antarctic prion feather *δ* ^15^N. Values are model predictions (mean ± SE) from a linear mixed-effects model with bird identity as a random intercept term (* *P* < 0.05, ** *P* < 0.01)

**Fig. 4:**
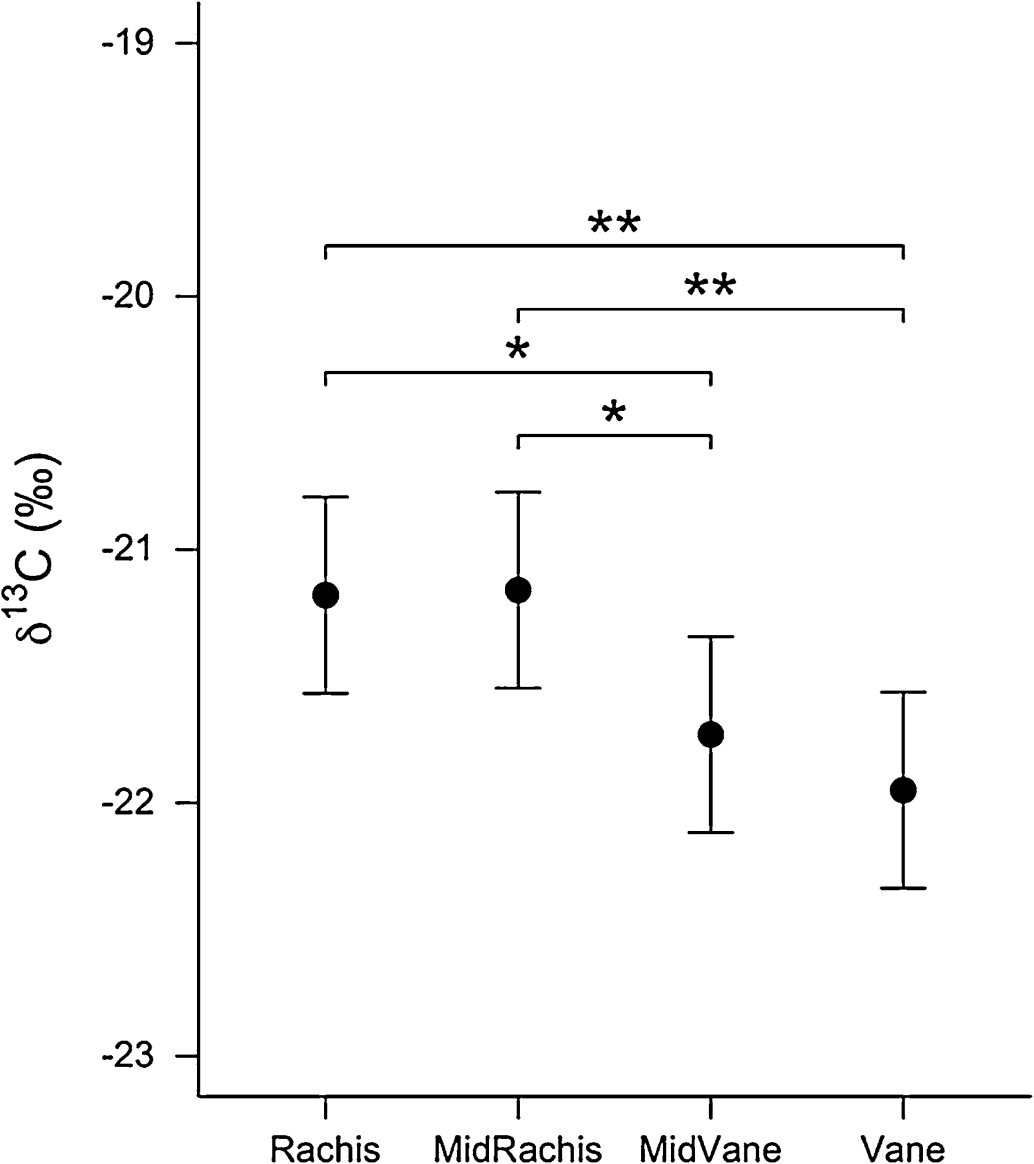
Post hoc comparison of the differences in Antarctic prion feather *δ* ^13^C. Values are model predictions (mean ± SE) from a linear mixed-effects model with bird identity as a random intercept term (* *P* < 0.05, ** *P* < 0.01)

Post hoc comparisons (based on differences between least-squares means) revealed that Antarctic prion feather material from the base of the rachis was enriched in ^15^N compared to that from the mid-rachis (Estimate = 0.44 ± 0.13, *t*_27_ = 3.31, *P* = 0.003), mid-vane (Estimate = 0.30 ± 0.13, *t*_27_ = 2.26, *P* = 0.032) and vane (Estimate = 0.36 ± 0.13, *t*_27_ = 2.71, *P* = 0.012; Fig. 3). Rachis material was also enriched in ^13^C relative to mid-vane (0.55 ± 0.24, *t*_27_ = 2.30, *P* = 0.029) and vane (0.77 ± 0.24, *t*_27_ = 3.22, *P* = 0.003) material (Fig. 4). Mid-rachis material was enriched in ^13^C relative to both the mid-vane (0.57 ± 0.24, *t*_27_ = 2.39, *P* = 0.024) and vane (0.79 ± 0.24, *t*_27_ = 3.31, *P* = 0.003; Fig. 3). See supplementary table.

### Sources of variation

In all cases, the proportion of variance explained by the random effects (range 20.3–75.3 %) was greater than that explained by the fixed effect of sample type (range 0.8–11.5 %). For the broad-billed prions, Bird ID explained more variation (54.2 %) than Feather ID (32.5 %) in *δ* ^15^N, whereas for *δ* ^13^C, Feather ID explained more variation (28.1 %) than Bird ID (20.3 %). For Antarctic prion samples, Bird ID fitted as a single random intercept explained a large amount of variation in both *δ* ^15^N (52.3 %) and *δ* ^13^C (75.3 %) of Antarctic prions.

## Discussion

In this study, we provide a detailed examination of isotopic variation in feather tissue from two species of prion. We detected within-feather differences in *δ* ^15^N but not *δ* ^13^C in primary (P2, P5 and P9) and primary covert feathers of broad-billed prions, and in both *δ* ^15^N and *δ* ^13^C in primary (P5) feathers of Antarctic prions. However, for both species, the proportion of variation explained by sample differences was small when compared to the influence of feather type and bird identity. The application of linear mixed-effects models to our data allows the partitioning of variance components and highlights the importance of considering variation both within and between feathers. We discuss the implications of variation in feather stable isotope values in terms of their application to studies of animal movement and diet.

### Variation in *δ*^13^C

We did not detect a difference in *δ* ^13^C between samples from the base and tip of broad-billed prion feathers, but this result was on the border of significance (*P* = 0.050). A large portion of variance in *δ* ^13^C was due to between-feather differences. Broad-billed prions are thought to begin moult during the postbreeding exodus in January and to moult primaries sequentially (i.e. P2 is replaced before P5 and then P9), before returning to the colony in February (Marchant and Higgins 1990). Many species of seabird move between distinct areas during the nonbreeding season (Phillips et al. 2005; González-Solís et al. 2011; Stenhouse et al. 2012), and movement within the period that feathers are being grown is likely to be reflected by shifts in *δ* ^13^C both within and between feathers. For example, through seasonal, productivity-related changes to *δ* ^13^C or variation in prey isotope values due to ontogenetic diet shifts in zooplankton such as copepods (Schmidt et al. 2003). The variation that we observed in prion feathers is therefore informative, as it may indicate changes in distribution or diet during feather regrowth (Thompson and Furness 1995).

Only one feather was available for each Antarctic prion included in the analysis, and so we could not test for between-feather differences, although recent work suggests large between-feather isotopic differences in this species (Carravieri et al. 2014). Within-feather comparisons suggested significant differences in *δ* ^13^C between rachis and vane samples but not within rachis and vane samples. The mid-rachis and mid-vane sections of a feather are presumably grown at the same time and so can be assumed to reflect the same dietary isotope assimilation. The significant difference in *δ* ^13^C but not *δ* ^15^N between these two samples therefore suggests a consistent structural difference between rachis and vane, and could be the result of different diet–tissue discrimination factors.

### Variation in *δ*^15^N

We detected within-feather differences in *δ* ^15^N for both species. Broad-billed prion feather vane differed from rachis material and, for Antarctic prion samples, mid-rachis and mid-vane did not differ in *δ* ^15^N, but rachis differed from mid-rachis, mid-vane and vane. This longitudinal difference in *δ* ^15^N within a feather could reflect changes in distribution or diet during feather growth. Despite these differences, when fitted as a random intercept, bird identity explained a larger proportion of variation in *δ* ^15^N than sample type in models for both species.

All six species of prion are very similar in appearance, but differences in bill morphology correspond to varying degrees of prey specialisation (Brooke 2004). Broad-billed prions have the most highly specialised bill, which is very large relative to the head and lined with lamellae that fringe the upper mandible to aid filter feeding on copepods (Klages and Cooper 1992; Brooke 2004). By comparison, the bill of an Antarctic prion is smaller and narrower, and this species is less reliant on copepods; their diet is dominated by crustacea although they are known to switch from krill *Euphausia* spp. to calanoid copepods in years of low krill abundance (Reid et al. 1997). Differences in *δ* ^15^N may therefore reflect prey switching, or baseline shifts in prey such as ontogenetic changes in the diet of copepods (Schmidt et al. 2003). Nevertheless, persistent among-individual differences in *δ* ^15^N may also be the result of individual specialisation (Bolnick et al. 2003; Ceia et al. 2012; Patrick et al. 2014).

### Sources of variation

During the synthesis of feather keratin, sulphur amino acids (SAAs) are vital exogenous nutrients due to the importance of sulphydryl bonds in maintaining keratin structure (Murphy and King 1982, 1984, 1987). Structurally similar sections of the feather, for example, the calamus and rachis, have similar amino acid compositions, whereas the barbs that make up the feather vane are structurally different and contain higher concentrations of SAAs (Schroeder et al. 1955; King and Murphy 1987; Murphy et al. 1990). Within-feather differences in *δ* ^13^C may therefore be due to differences in amino acid composition and suggest that storage of SAAs ingested previously may affect subsequent isotopic values within the constituent parts of individual feathers. This interpretation invites compound-specific stable isotope analysis to investigate the role of specific amino acids in feather synthesis. Furthermore, while variation in the SAA composition of feathers among individuals is thought to be small within species (Murphy and King 1982), there may be differences between species (Schroeder et al. 1955; Harrap and Woods 1967).

The physiological or nutritional status of an individual may present an additional source of isotopic variation (Cherel et al. 2005; Sears et al. 2009), and isotopic discrimination rates can differ between species (Hobson and Clark 1992b). Small effects of growth or diet restriction on isotope values may be obscured by the effect of changes in prey isotope signatures related to a change in diet or distribution during the moulting period (Sears et al. 2009). In addition, some seabird species begin partial feather moult at the breeding grounds, but then suspend it until they reach the nonbreeding area (Ramos et al. 2009b; Catry et al. 2013). Moult-breeding overlap is more common in species or populations that are predominantly sedentary (Bridge 2006), and so isotope values in some feathers may reflect prey consumed while around the colony. Within-feather isotopic variation may also vary as a function of feather moult rate, for example, large seabirds that grow feathers over extended periods (Rohwer et al. 2009) are more likely to move part-way through the growth period and so may incorporate isotopic shifts in their feathers.

### Implications for future isotope studies

Isotopic variation in body tissues is informative when it can be used to indicate changes in location or diet during the period of feather moult and regrowth (Thompson and Furness 1995; Ramos et al. 2009a; González-Solís et al. 2011). However, it is important to consider whether within-feather differences are ecologically meaningful (Authier et al. 2012). In this study, variation in *δ* ^13^C and *δ* ^15^N indicates that between-feather differences in *δ* ^13^C and between-individual differences in *δ* ^15^N may be more important than within-feather differences. Nevertheless, it is important to note that the magnitude of within-feather differences was up to ~0.5 ‰ in *δ* ^15^N and up to ~0.8 ‰ in *δ* ^13^C after accounting for individual and feather differences, and so could influence interpretation if part of a feather was sampled in isolation.

Sampling strategies that include multiple samples from an individual or particular feather could account for this type of isotopic variation, while also allowing potential within-individual differences to be examined (Jaeger et al. 2009). Nevertheless, future studies should be cautious when using different sub-sections of a feather and avoid comparisons between rachis and vane material. Alternatively, sampling protocols could include material from all parts of the feather, either by sampling along the feather (Wiley et al. 2010), or by grinding whole feathers (Moreno et al. 2011). These methods could prevent isotopic differences from single feather samples being interpreted as reflecting either among-individual diet specialisation or differences in winter strategies when they may instead simply relate to within-feather differences.

### Summary

In summary, detailed examination of isotopic variation in feather constituents revealed: (1) individual-level differences in *δ* ^15^N attributed to dietary differences; (2) between-feather differences in *δ* ^13^C probably resulting from movement during moult; (3) within-feather differences in *δ* ^15^N attributed to temporal dietary shifts during feather regrowth; and (4) within-feather differences in *δ* ^13^C attributed to structural differences within feathers. Although the present study does not explore the mechanisms that explain the differences we observe, techniques such as compound-specific isotope analysis would allow differences in the amino acid composition of feathers to be considered, or the origin of particular compounds to be discerned (Lorrain et al. 2009). Finally, while single feather analysis can provide information on dietary differences between individuals, future studies should consider sampling strategies that allow potential within-individual differences to be examined.

## Electronic supplementary material

Below is the link to the electronic supplementary material.

Supplementary material 1 (PDF 82 kb)
