# Supplementary material for: Quantifying variation in δ13C and δ15N isotopes within and between feathers and individuals: Is one sample enough?
Source: Mar Biol. 2015 Feb 15;162(4):733–41. doi: 10.1007/s00227-015-2618-8 (PMC4356738; doi:10.1007/s00227-015-2618-8)
Supplement: Supplementary file 1 — Supplementary material 1 (PDF 82 kb) [file 227_2015_2618_MOESM1_ESM.pdf]

Quantifying variation in δ^{13}C and δ^{15}N isotopes within and between feathers and individuals; is one sample enough?

Marine Biology

W. James Grecian[1*], Rona A. R. McGill[2], Richard A. Phillips[3], Peter G. Ryan[4] & Robert W. Furness[1]

[1] Institute of Biodiversity, Animal Health & Comparative Medicine, College of Medical, Veterinary & Life Sciences, Graham Kerr Building, University of Glasgow, Glasgow, G12 8QQ, U.K.

[2] NERC Life Sciences Mass Spectrometry Facility,
Scottish Universities Environmental Research Centre, Rankine Avenue,
East Kilbride, G75 0QF, U.K.

[3] British Antarctic Survey, Natural Environment Research Council, High Cross, Madingley Road, Cambridge CB3 0ET, U.K.

[4] Percy FitzPatrick Institute, DST/NRF Centre of Excellence, University of Cape Town, Rondebosch 7701, South Africa

[*] Corresponding author: james.grecian@glasgow.ac.uk

Supplementary material

Post-hoc comparison of the differences in stable isotope values between Antarctic prion feather material samples. Estimates calculated using differences in least squares means with Satterthwaite's approximation for degrees of freedom from linear mixed-effects models with bird identity as a random intercept term. Data is presented in Figures 2 and 3, bold highlights significant differences.

**Sample Interaction**	**Est. δ^{15}N ± SE**	**CIs**	**t_{27}**	***P***
**Rachis : Mid-Rachis**	**0.44 ± 0.13**	**0.17 - 0.71**	**3.31**	**0.002**
**Rachis : Mid-Vane**	**0.30 ± 0.13**	**0.03 - 0.57**	**2.26**	**0.032**
**Rachis : Vane**	**0.36 ± 0.13**	**0.09 - 0.63**	**2.71**	**0.012**
Mid-Rachis : Mid-Vane	-0.14 ± 0.13	-0.41 - 0.13	-1.05	0.301
Mid-Rachis : Vane	-0.08 ± 0.13	-0.35 - 0.19	-0.60	0.552
Mid-Vane : Vane	0.06 ± 0.13	-0.21 - 0.33	0.45	0.655

**Sample Interaction**	**Est. δ^{13}C ± SE**	**CIs**	**t_{27}**	***P***
Rachis : Mid-Rachis	-0.02 ± 0.24	-0.51 - 0.47	-0.08	0.934
**Rachis : Mid-Vane**	**0.55 ± 0.24**	**0.06 - 1.04**	**2.30**	**0.029**
**Rachis : Vane**	**0.77 ± 0.24**	**0.28 - 1.26**	**3.22**	**0.003**
**Mid-Rachis : Mid-Vane**	**0.57 ± 0.24**	**0.08 - 1.06**	**2.39**	**0.024**
**Mid-Rachis : Vane**	**0.79 ± 0.24**	**0.30 - 1.28**	**3.31**	**0.003**
Mid-Vane : Vane	0.22 ± 0.24	-0.27 - 0.71	0.92	0.365
